# Antiobesity Effects of an Edible Halophyte *Nitraria retusa* Forssk in 3T3-L1 Preadipocyte Differentiation and in C57B6J/L Mice Fed a High Fat Diet-Induced Obesity

**DOI:** 10.1155/2013/368658

**Published:** 2013-12-03

**Authors:** Feten Zar Kalai, Junkyu Han, Riadh Ksouri, Abdelfatteh El Omri, Chedly Abdelly, Hiroko Isoda

**Affiliations:** ^1^Graduate School of Life and Environmental Sciences, University of Tsukuba, Tsukuba, Ibaraki 305-8572, Japan; ^2^Faculty of Life and Environmental Sciences, University of Tsukuba, Tsukuba, Ibaraki 305-8572, Japan; ^3^Alliance for Research on North Africa (ARENA), University of Tsukuba, Tsukuba, Ibaraki 305-8572, Japan; ^4^Laboratoire des Plantes Extrêmophiles, Centre de Biotechnologie à la Technopole de BorjCédria (CBBC), BP 901, 2050 Hammam-Lif, Tunisia

## Abstract

*Nitraria retusa* is an edible halophyte, used in Tunisia for several traditional medicine purposes. The present study investigated the antiobesity effects of *Nitraria retusa* ethanol extract (NRE) in 3T3-L1 cells using different doses and in high-fat diet-induced obesity in mice. Male C57B6J/L mice were separately fed a normal diet (ND) or a high-fat diet (HFD) and daily administrated with NRE (50, 100 mg/kg) or one for 2 days with Naringenin (10 mg/kg). NRE administration significantly decreased body weight gain, fat pad weight, serum glucose, and lipid levels in HFD-induced obese mice. To elucidate the mechanism of action of NRE, the expression of genes involved in lipid and carbohydrate metabolism were measured in liver. Results showed that mice treated with NRE demonstrated a significant decrease in cumulative body weight and fat pad weight, a significant lowering in glucose and triglycerides serum levels, and an increase in the HDL-cholesterol serum level. Moreover mRNA expression results showed an enhancement of the expression of genes related to liver metabolism. Our findings suggest that NRE treatment had a protective or controlling effect against a high fat diet-induced obesity in C57B6J/L mice through the regulation of expression of genes involved in lipolysis and lipogenesis and thus the enhancement of the lipid metabolism in liver.

## 1. Introduction

Obesity is a rapidly growing epidemic worldwide, presenting an increase in the risk of morbidity and mortality in many countries across the world [1]. It has become an increasingly prevalent public health problem and represents the complex interaction of genetic, developmental, behavioral, and environmental influences [2]. The World Health Organization (WHO) defines obesity as an abnormal excessive fat accumulation detrimental to human health. Obesity has also been defined as an increased adipose tissue mass, which is the result of an enlargement in fat cells and/or an increase in their number [3]. Moreover, obesity is fundamentally a problem of energy balance in that self-evidently it can develop when energy intake exceeds energy expenditure, resulting in fat accumulation and excessive adipose tissue mass. Adipose tissue, in addition to its function as the major storage depot for triglycerides, is an active endocrine tissue sensing metabolic signals and secreting hormones called adipocytokines that affect whole-body energy homeostasis [4]. Since it is an endocrine organ, it has a fundamental role in metabolism and homeostasis regulation, where numerous chemical messengers called adipokines are released for better communication. The production and secretion of an excess or insufficient amount of adipokines may provide a molecular link between increased adiposity and the development of diabetes mellitus, metabolic syndrome, and cardiovascular diseases [5]. The main metabolic fuels of the body are glucose, fatty acids, and ketone bodies. In the metabolic homeostasis of the body as a whole, the liver occupies a central position. Indeed, besides building up glycogen in its own cells, the liver plays an essential role in the synthesis of adipose tissue triglycerides, by producing very low-density lipoproteins. Furthermore, the liver furnishes oxidizable substrates, not only to meet its own needs, but also to cover those of other tissues [6]. Besides, it has been reported that the liver plays an important role in modulating western diet-associated metabolic disorders. High-fat diets significantly alter the expression of many genes related to lipid, cholesterol, and oxidoreductive metabolism [7]. Nowadays, diets high in fats tend to promote obesity; hence inhibition of digestion and absorption of dietary fats are a biological remedy in treating obesity [8]. As synthetic drugs fail to give desired effects and with side effects involved, the utilization of traditional and alternative medicines is fast gaining acceptance. Medicinal plants are believed to harbor potential antiobesity agents that can act through various mechanisms either by preventing weight gain or promoting weight loss amongst them and this may be an excellent alternative strategy for developing future effective, safe antiobesity drugs [9]. Thus, the clinical importance of herbal drugs and polyphenols for treatment of obesity has received considerable attention [3]. These therapies have been variably efficacious on adipocyte differentiation and lipid accumulation in adipocytes. A number of herbal and dietary inhibitors of adipose differentiation have been identified, including isorhamnetin [10], Epigallocatechin-Gallate [11], quercetin [12], and Naringenin [13]. This family of phenolic compounds are potent scavengers of free radicals and potentially useful in the prevention of cancer and arteriosclerosis and also have been associated with several health promoting activities such as decreasing blood sugar levels and reducing body weight [14]. Halophytes are a salt-tolerant species from salt and arid regions and desert that can tolerate a wide range of environmental conditions and resist abiotic stresses such as salt, high temperature and luminosity, and drought stresses [15]. In fact, able to withstand and quench these severe environmental stresses, halophytes are equipped with powerful antioxidant systems that constitute mainly on phenolic compounds so-called “stress metabolites.” These plants have ethnopharmacological data indicating their utilization in folk medicine. Thus, the role of these medicinal species in the prevention or treatment of diseases has been largely attributed to their antioxidant properties associated with a wide range of bioactive molecules [16]. *Nitraria retusa* is one of the native perennial halophyte species that belong to the botanical family Nitrariaceae. It is distributed in North Africa and restricted to Algeria and Tunisia. In Tunisia, it is widespread in central and south parts. This salt-tolerant and drought resistant shrub grows along shallow and hummocks on saline grounds near the coastal areas and produces fleshy red fruits from which a tasty and refreshing juice may be extracted. *Nitraria retusa* is known in Tunisia as “Ghardaq.” The sweet drupes are edible for the treatment of hypertension. Leaves infusion and decoction are used as tea or cataplasm for their anti-inflammatory properties [17]. In previous chemosystematic investigation, the flavonoids contained in *Nitraria retusa* leaves were studied; six isorhamnetin glycosides (isorhamnetin 3-robinobioside, isorhamnetin 3-rutinoside, isorhamnetin-3-O-galactoside, isorhamnetin-3-o-glucoside, isorhamnetin 3-xylosylrobinobioside, and isorhamnetin-3-O-4Rhamgalactosylrobinobioside) and free isorhamnetin were identified [18]. These bioactive molecules isolated from *Nitraria retusa* have been reported to promote apoptosis in human myelogenous erythroleukemia cells [19] and to exert antioxidant and antimutagenic activities [20]. To the best of our knowledge, this is the first time to report the effect of *Nitraria retusa* ethanol extract (NRE) on inhibiting preadipocyte differentiation and lipid droplet accumulation in 3T3-L1 cells and reducing body weight in mice fed with high-fat diet in correlation with lipid metabolism in liver.

## 2. Materials and Methods

### 2.1. Plant Sampling

*Nitraria retusa* shoots were collected during August 2010 from the salt flat “Sabkha El Kelbia” located at N 35 48 44, E 10 09 06 (Kairouan, Tunisia). This locality is characterized by a semiarid climate with less rainfall <200 mm/year and higher salinity mean (20 g/L). The collected samples were rinsed with distilled water, kept in laboratory temperature, oven dried at 60°C, and then ground finely using a ball mill type “Dangoumeau.” The plant powder obtained was stored at room temperature for further experiments.

### 2.2. Extraction Methods

Seventy percent ethanol extraction *Nitraria retusa* sample was conducted with 10% (w/v). The ethanol extract was kept in the dark at room temperature for 2 weeks, with shaking at least once a day. The liquid fraction was then collected, filtered through 0.22 *μ*m filter (MILLIPORE, U.S.A.), and concentrated using SpeedVac (SCRUM Inc., Japan). The dried residue was redissolved in seventy percent ethanol or milli-Q water by vortexing and stored at −80°C for further experiments.

### 2.3. Cell Culture

Murine 3T3-L1 preadipocytes (Riken Tsukuba, Japan) were maintained in Dulbecco's Modified Eagle's Medium (DMEM) supplemented with 10% fetal bovine serum (FBS) and 1% penicillin (5000 *μ*g/mL)-streptomycin (5000 IU/mL) in 75 cm^2^ tissue culture flasks. Medium was changed every 3 days and cell passage was carried out at 80% confluence at one on two ratio using 0.25% trypsin (1 mM EDTA). 3T3-L1 cells were cultured in a humidified incubator at 37°C and 5% CO_2_.

### 2.4. Preadipocytes Differentiation and Oil Red-O Staining Procedures

3T3-L1 preadipocytes were seeded into 96-well plates at 1.0 × 10^4^ cell/well and cultured for additional two days until full confluence. Two days later (day 0), cells were incubated with a differentiation cocktail (MDI) containing 1/10 insulin solution, 1/10 dexamethasone solution, and 1/10 3-isobutyl-1-methylxanthine solution in standard culture medium for 3 days followed by additional 48 h with standard culture medium containing insulin alone. The differentiation-maintenance medium was changed every 2 days. To investigate the effect of *Nitraria retusa* on adipogenesis in 3T3-L1, NRE (25, 50, 100, 200, and 400 *μ*g/mL) was added to the differentiation-induction and differentiation-maintenance media. The staining procedure was conducted according to the adipogenesis assay kit (Cayman Chemical Company). The absorbance was read at 490 nm with a 96-well plate reader. The lipid droplet content was reported as percentage of control cells, and isorhamnetin-treated cells were used as positive control.

### 2.5. Animals and Experimental Design

Four-week-old, male C57B6/JL mice were purchased from Charles River (Japan) and were maintained under a light cycle (12 h light/dark) and fed with a high-fat diet (HFD) or a normal diet (ND), purchased from Oriental Yeast Company (Japan) and according to the composition described in Table 1. After 1 week acclimatization, mice were divided randomly into 5 groups with 8 individuals for each group: control normal diet group (CND) fed with normal diet and orally administrated with water as vehicle, control high-fat diet group fed with HFD and orally administrated with water as vehicle (CHFD), high-fat diet group fed with HFD and orally administrated with Naringenin as positive control at a dose of 10 mg/kg of body weight (HFD + PC), high-fat diet group fed with HFD and orally administrated with *Nitraria retusa* at 50 mg/kg of body weight (HFD + NR50), and high-fat diet group fed with HFD and orally administrated with NRE at 100 mg/kg of body weight (HFD + NR100). Body weight and food intakes were measured daily at regular intervals during the feeding period (28 days). Following 4 weeks treatment, animals were sacrificed. Blood samples were collected and liver and fat tissue were dissected, weighed, and kept at −80°C until use. All procedures were performed in accordance with the Ethics Animal Care and Use Committee of the University of Tsukuba, Japan.

### 2.6. Biochemical Analysis of Blood Serum Analysis

Collected blood samples were centrifuged at 3,000 rpm for 15 min at 4°C. Then several metabolites like serum glucose, triglyceride (TG), total cholesterol (TCHO), high-density lipoprotein (HDL), and low-density lipoprotein (LDL) levels were measured according to the kit manufacturer's instruction. The cytokine tumor necrosis factor-alpha (TNF-*α*) level in serum was also analyzed using the enzyme linked immunosorbent assay (ELISA) (Invitrogen Ms TNF-*α* kit) according to the manufacturer's instructions.

### 2.7. RNA Isolation from Liver and Real-Time PCR Analysis

50 mg liver samples were homogenized using Polytron PT 1200 E homogenizer (Switzerland). Then total RNA was purified using the ISOGEN kit (Nippon GeneCo. Ltd., Japan) following the manufacturer's instructions. Total RNA was quantified and quality-checked using Thermo Scientific NanoDrop 2000 (USA) and the reverse transcription reactions were performed using the Superscript III reverse transcriptase kit (Invitrogen, Carlsbad/CA, USA) using 1 *μ*g of total RNA. Briefly, RNA was denatured by incubation at 65°C for 5 min, with 1 *μ*L oligo (dT) primers, and chilled at 4°C. Then SuperScript III reverse transcriptase was added and the reaction mix was then incubated at 42°C for 60 min and then 10 min at 70°C [21]. The gene expression of peroxisome proliferator-activated receptor gamma 1 (PPAR *γ* 1), peroxisome proliferator-activated receptor alpha (PPAR *α*), lipoprotein lipase (LPL), fatty acid synthase (FAS), Acetyl-CoA Carboxylase 1 (ACC1), and carnitine palmitoyl transferase I (CPT1) were determined by real-time PCR, normalized to beta-actin, and reported as fold of control. Primers and TaqMan probes used for these experiments were purchased from Applied Biosystems. Primers were inventoried gene expression assays. TaqMan real-time PCR amplification reactions were performed in a 20 *μ*L reaction mixtures containing 10 *μ*L of TaqMan Universal PCR Master Mix UNG (2X), 9 *μ*L of template cDNA (100 ng/*μ*L), and 1 *μ*L of the corresponding primer/probe mix, using an AB 7500 fast real-time system (Applied Biosystems). For the amplification, the following cycling conditions were applied: 2 min at 50°C, 10 min at 95°C, and 40 cycles of 15 s at 95°C/1 min at 60°C.

## 3. Statistical Analysis

All experiments were repeated at least three times. Data were expressed as the mean ± SD or the mean ± SEM. Differences between control and treatments were assessed by Student's unpaired *t*-test. *P* values below 0.05, 0.005, and 0.001 were considered significant.

## 4. Results

### 4.1. NRE Reduces Cell Differentiation and Lipid Droplet Formation in 3T3-L1 Cells

**Figure 1:**
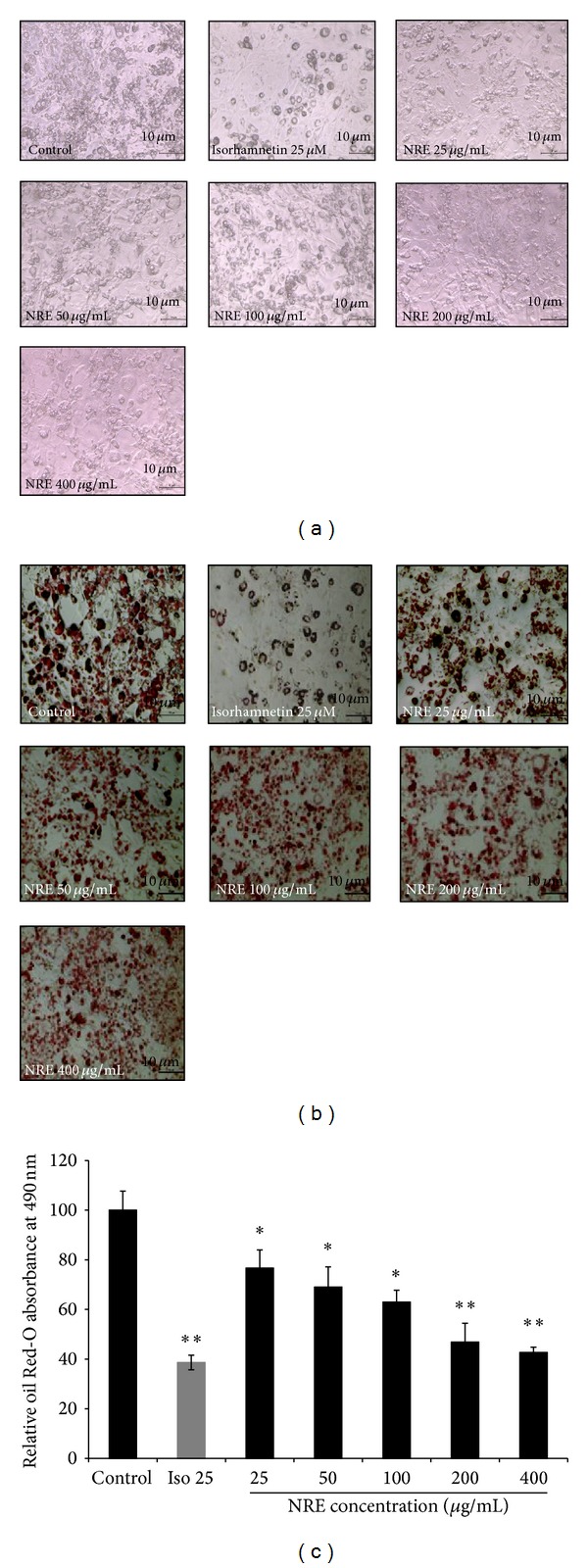
Effects of *Nitraria retusa *(NR) extract using different concentrations (25, 50, 100, 200, and 400 *μ*g/mL) on cell differentiation and fat droplet formation in 3T3-L1 cells before (a) and after (b) oil Red-O staining. Fat droplets in preadipocytes and adipocytes differentiated for 9 days with or without *Nitraria retusa* (NR) and isorhamnetin (the positive control) treatments were stained with oil Red-O dye and examined using a light microscope. (c) Effects of *Nitraria retusa* extract NR on lipid droplet content in 3T3-L1 cells. Lipid droplet accumulation in treated cells was expressed as a percentage of control (untreated cells). Bars represent mean ± SD, *n* = 3, *P < 0.05, **P < 0.005.

Adipogenesis assay was performed to investigate the effect of NRE on the adipocyte differentiation and on the lipid droplets accumulation in 3T3-L1 cells using oil Red-O staining. Differentiated 3T3-L1 cells were treated every two days with NRE at various concentration and with 25 *μ*M isorhamnetin (as a positive control), for 7 days. Based on oil Red-O content quantification, results showed that NRE treatment at 25, 50, 100, 200, and 400 *μ*g/mL in 3T3-L1 cells could inhibit the lipid droplet accumulation compared to untreated cells, in dose dependent manner (Figure 1(c)). The triglyceride accumulation significantly decreased to 76.60 ± 7.30%, 69.08 ± 8.08%, 62.90 ± 4.80%, 46.80 ± 7.50%, and 42.70 ± 2.10%, respectively (Figure 1(c)), without any cytotoxic effect (data not shown). At 200 and 400 *μ*g/mL, NRE treatment showed similar effect as isorhamnetin. Moreover, we noticed that NRE treatment in 3T3-L1 was accompanied by modulation of cell hypertrophy rather than cell hyperplasia as indicated in microscopic observation (Figures 1(a) and 1(b)). Thus NRE treatment might induce the cell differentiation into smaller adipocytes compared to untreated cells.

### 4.2. Antiobesity Effects of NRE in HFD-Induced C57B6J/L Obese Mice

**Figure 2:**
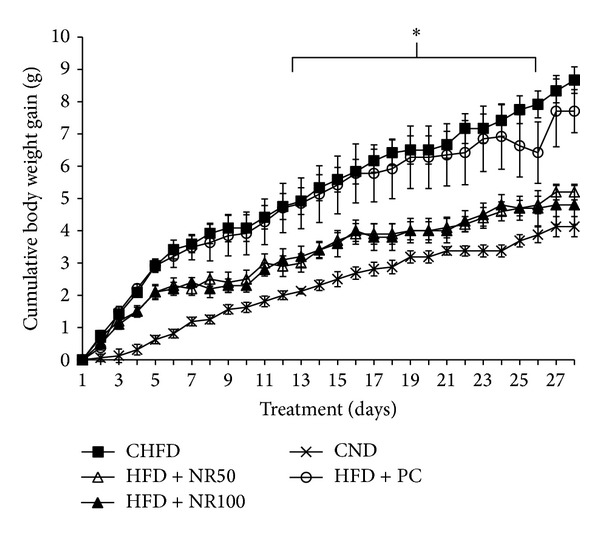
Effect of daily oral administration of *Nitraria retusa* (50 and 100 mg/kg body weight) on cumulative body weight gain of mice fed with high-fat diet. Mice were fed normal diet (CND), high-fat diet (CHFD), high-fat diet supplemented with positive control Naringenin 10 mg/kg body weight (HFD + PC), high-fat diet supplemented with *Nitraria retusa* 50 mg/kg body weight (HFD + NR50), or high-fat diet supplemented with *Nitraria retusa* 100 mg/kg body weight (HFD + NR100) for 4 weeks. Body weight was daily measured at regular time. Data represent mean ± SEM, *n* = 8, *P < 0.05, **P < 0.005 compared to the high-fat diet group.

**Figure 3:**
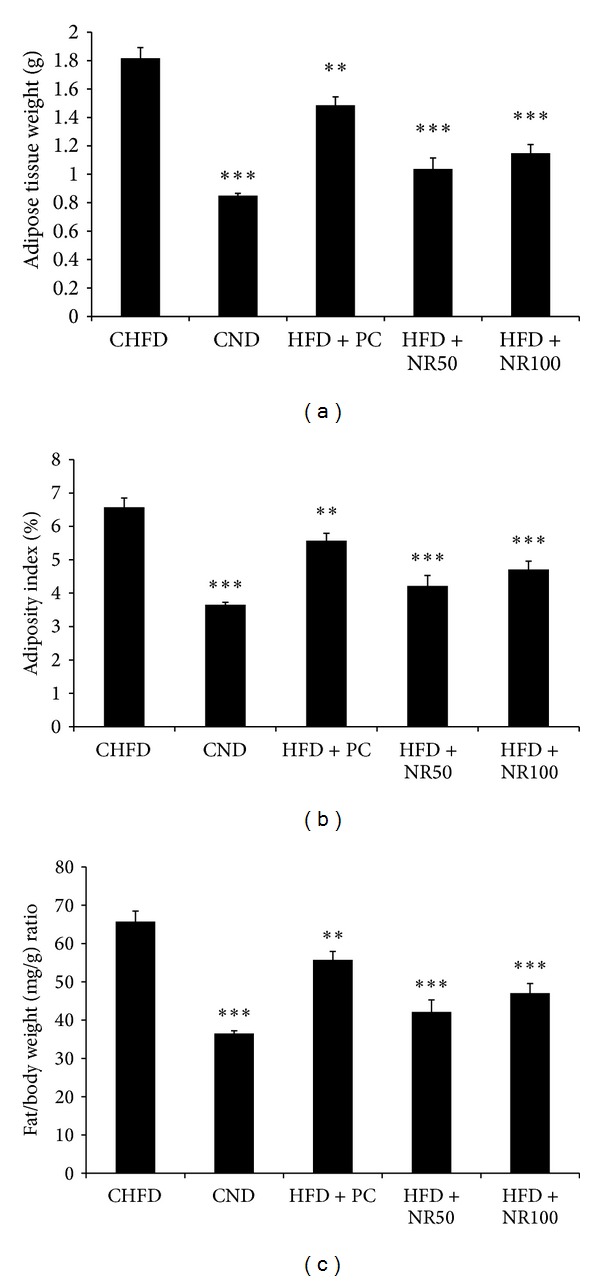
Effect of daily oral administration of *Nitraria retusa* (50 and 100 mg/kg body weight) on adipose tissue weight (a), adiposity index (b), and fat/body weight (mg/g) ratio (c) of mice fed with high-fat diet. Mice were fed normal diet (CND), high-fat diet (CHFD), high-fat diet supplemented with positive control Naringenin 10 mg/kg body weight (HFD + PC), high-fat diet supplemented with *Nitraria retusa* 50 mg/kg body weight (HFD + NR50), or high-fat diet supplemented with *Nitraria retusa* 100 mg/kg body weight (HFD + NR100) for 4 weeks. At the end of experiment, adipose tissue, for all groups, was weighed. Data represent mean ± SEM, *n* = 8, *P < 0.05, **P < 0.005, ***P < 0.001 compared to the high-fat diet group.

As shown in Figure 2, the HFD increased body weight gain significantly compared to ND over 4 weeks treatment period in C57B6J/L mice. Moreover, final body weight was significantly lower in the HFD + NR50 (24.60 ± 0.50 g) and HFD + NR100 (24.40 ± 0.70 g) groups compared to CHFD group (27.62 ± 0.50 g) (Table 2) (*P* < 0.05), without affecting food intake, which was around 3 g/day/mice for all different experimental groups (Table 3). It is well known that body weight and fat stores are determined by the net excess or deficit of food intake over energy expenditure. In the current study, NRE treatment was demonstrated not only to decrease cumulative body weight gain but also adipose tissue weight and improve adiposity index. In fact 50 mg/kg and 100 mg/kg NRE treatment in HFD mice decreased adipose tissue weight from 1.80 ± 0.20 g in vehicle group to 1.10 ± 0.21 g and 1.20 ± 0.16 g, respectively (Figure 3).

### 4.3. Effect of NRE Administration on Glucose, Triglycerides, Cholesterol, and TNF-*α* in HFD-Induced C57B6/JL Obese Mice

Serum glucose and lipid levels (triglycerides, total cholesterol, HDL-cholesterol (HDL-c), and LDL-cholesterol (LDL-c)) and also the cytokine TNF-*α* level of all mice groups were analyzed. Results are summarized in Table 4. The HFD-fed mice showed significant high levels of serum glucose (200.00 ± 4.10 mg/dL), TG (22.00 ± 1.63 mg/dL), and lower level of HDL-c (80.00 ± 1.55 mg/dL) when compared to those fed with normal diet (CND group). As shown in Table 4, 50 mg/kg and 100 mg/kg NRE and Naringenin (positive control) treatments significantly decreased glucose levels in blood serum of HFD-fed mice to reach 168.00 ± 2.69 mg/dL, 153.00 ± 9.41 mg/dL, and 146.00 ± 1.63 mg/dL, respectively. Triglycerides levels were also significantly decreased to 10.00 ± 1.63 mg/dL and 14.00 ± 1.63 mg/dL in 50 mg/kg and 100 mg/kg NRE treated HFD mice, respectively. However, Naringenin treatment did not affect their levels. NRE administration for 4 weeks did not affect the total cholesterol in HFD-induced obese mice blood serum. However, it significantly increased HDL-c fraction (the good cholesterol) from 80.00 ± 1.55 mg/dL in vehicle group to 98.00 ± 4.10 mg/dL, and 92.00 ± 4.10 mg/dL, respectively, for HFD + NR50 and HFD + 100 groups (*P* < 0.05). High-density lipoprotein (HDL) particles transport cholesterol back to the liver for excretion, but vary considerably in their effectiveness for doing this. Having large numbers of large HDL particles correlates with better health outcomes, and hence it is commonly called “good cholesterol.” On the other hand, only 100 mg/kg NRE administration, significantly, decreased LDL-c (the bad cholesterol) from 16.50 ± 2.03 mg/dL for vehicle group to 12.00 mg/dL (*P* < 0.05).

### 4.4. Effect of NRE Treatment on Hepatic Lipid Metabolism Gene Expression in HFD-Induced C57B6/JL Obese Mice

High serum lipid level mainly triglycerides and cholesterol are a hallmark of many metabolic syndrome diseases such as type 2 diabetes. Understanding the molecular mechanism that undergoes dyslipidemia should facilitate improved strategies for serum lipid management. In this respect, the effect of NRE administration in HFD-induced mice on hepatic lipid metabolism biomarkers at translational level was investigated, following 4 weeks experimental study.

**Figure 4:**
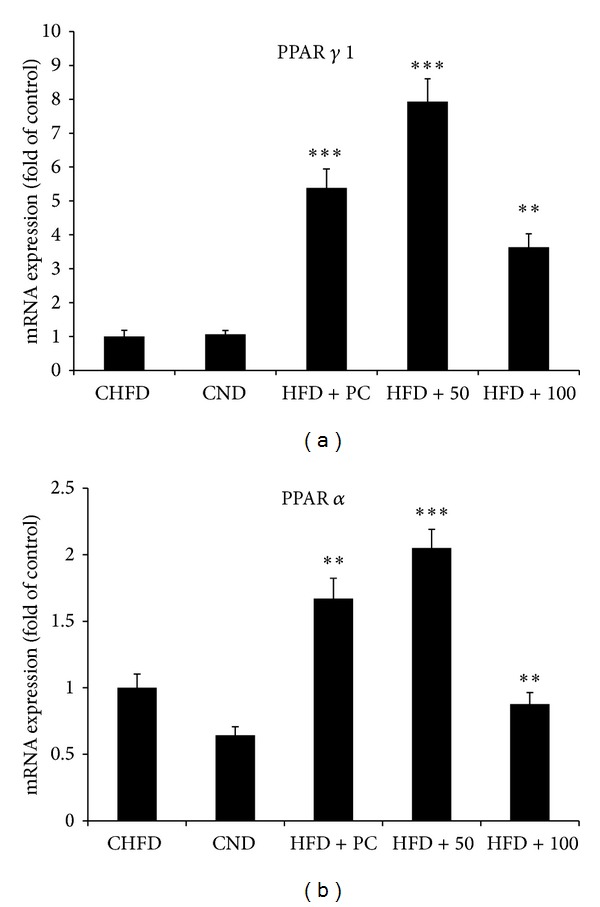
Effect of daily oral administration of *Nitraria retusa* (50 and 100 mg/kg body weight) on genes regulating lipid metabolism in liver of mice fed with high-fat diet. Peroxisome proliferator activated receptor gamma (PPAR *γ* 1) (a) and peroxisome proliferator activated receptor alpha (PPAR *α*) (b). Real-time PCR was conducted and result was expressed as mRNA expression fold change compared to the control high-fat diet (HFD). Bars represent mean ± SD. *P < 0.05, **P < 0.005 compared to the high-fat diet group.

**Figure 5:**
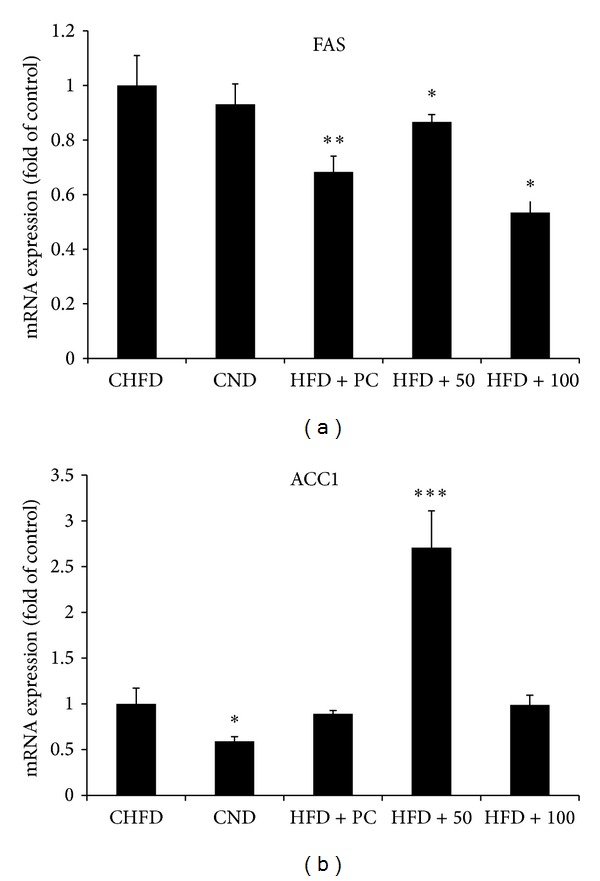
Effect of daily oral administration of *Nitraria retusa* (50 and 100 mg/kg body weight) on genes regulating lipid metabolism in liver of mice fed with high-fat diet. Lipogenic enzymes; fatty acid synthase (FAS) (a) and Acetyl-CoA Carboxylase 1 (ACC1) (b). Real-time PCR was conducted and result was expressed as mRNA expression fold change compared to the control high-fat diet (HFD). Bars represent mean ± SD. *P < 0.05, **P < 0.005 compared to the high-fat diet group.

**Figure 6:**
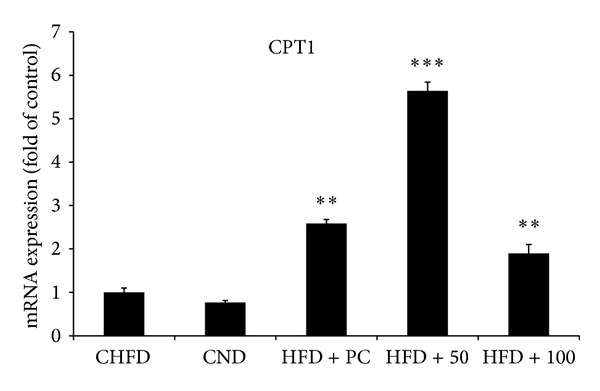
Effect of daily oral administration of *Nitraria retusa* (50 and 100 mg/kg body weight) on genes regulating lipid metabolism in liver of mice fed with high-fat diet. Carnitine palmitoyltransferase I (CPT1) essential step in the beta-oxidation of long chain fatty acids. Real-time PCR was conducted and result was expressed as mRNA expression fold change compared to the control high-fat diet (HFD). Bars represent mean ± SD. *P < 0.05, **P < 0.005 compared to the high-fat diet group.

**Figure 7:**
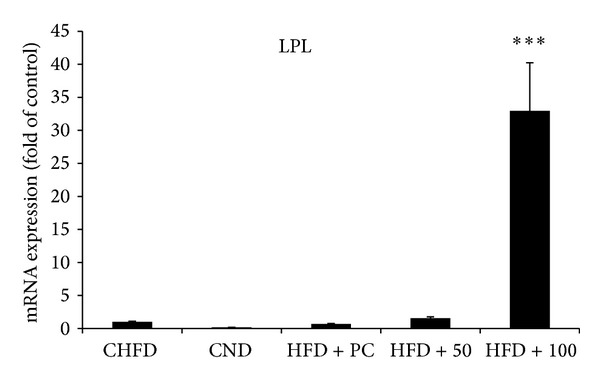
Effect of daily oral administration of *Nitraria retusa* (50 and 100 mg/kg body weight) on genes regulating lipid metabolism in liver of mice fed with high-fat diet. Lipoprotein lipase (LPL) enzyme responsible for the hydrolysis of triglycerides in lipoproteins. Real-time PCR was conducted and result was expressed as mRNA expression fold change compared to the control high-fat diet (HFD). Bars represent mean ± SD. *P < 0.05, **P < 0.005 compared to the high-fat diet group.

In fact, 50 mg/kg and 100 mg/kg NRE treatments significantly increased the gene expression of the hepatic PPAR *γ* 1 (Figure 4(a)) to 6 and 4 folds, respectively. While only 50 mg/kg NRE dose improved PPAR *α* gene expression (Figure 4(b)) by 2 folds. Moreover, NRE treatments significantly modulated the lipogenic enzyme genes ACC1 and FAS. In fact FAS gene expression decreased by half at 100 mg/kg dose (Figure 5(a)), and ACC1 gene expression increased by 2.5 folds at 50 mg/kg dose (Figure 5(b)). Additionally, 50 mg/kg NRE treatment in HFD-induced obese C57BL/6J mice significantly increased CPT1 (Figure 6) and LPL (Figure 7) gene expression to reach 6 and 30 folds, respectively. These results were concordant with weight loss, adiposity index, and biochemical metabolites investigation. The current data indicates that *in vivo* administration of NRE could be an effective plant preparation in enhancing liver lipid metabolism and preventing obesity.

## 5. Discussion

In the current study, NRE treatment in differentiated adipocyte 3T3-L1 cells significantly inhibited lipid droplet accumulation and modulated cell hypertrophy and not cell hyperplasia in a similar trend as isorhamnetin treatment. In fact, the adipocyte is the primary site of energy storage and triglycerides accumulation during nutritional excess. Moreover, NRE administrations in HFD-induced obese C57BL/6J mice for 4 consecutive weeks significantly reduced body weight gain and adipose tissue accumulation without affecting food intake. Furthermore, these effects were in concordance with a significant improvement of glucose and lipid metabolism in blood serum and the expression profiles of genes related to beta-oxidation, lipolysis, and lipogenesis in the liver. These findings demonstrated that NRE suppresses obesity in HFD-induced obese mice.

It is well known that obesity is caused by imbalanced homeostasis between low energy expenditure and increased energy intake and accumulation [22]. Excess energy is mainly stored as triglycerides in adipose tissue which increase the visceral adipose tissue mass through adipocyte hypertrophy and hyperplasia. Several strategies are proposed to reduce or suppress obesity, among them dietary supplements and natural products. In fact, herbal and botanical preparations are gaining a lot of interest either to substitute chemical drugs or to be combined with them. In this respect, *Nitraria retusa*, an edible halophyte plant growing wild in Tunisia, could be a potent candidate. Previous phytochemical studies from our research and others demonstrated that *Nitraria retusa *has a strong anti-oxidant and free radical scavenging properties due mainly to its high contents in polyphenols and flavonoids [23]. The HPLC analysis showed the presence of several alkaloids like 5, 7-dihydroxy-3-deoxy-vasiciene I, 7-hydroxy-3-deoxy1-vasiciene II, and O-acetylnitraraine I [18]. The phenolic profile showed mainly, high contents of isorhamnetin aglycone and glycosides [23] and other flavonoids like apigenin, quercetin, kaempferol, and luteolin [24]. The high content in flavonoids and their possible synergetic effect may explain in part the antiobesity effect of NRE, since these compounds were individually demonstrated to have high potential to prevent metabolic syndrome diseases [25] and their mixture showed synergetic antiobesity effect [26–33].

The investigation of biochemical markers like glucose, total triglycerides, total cholesterol, LDL, HDL, and TNF-*α* in mice blood serum demonstrated that NRE significantly improved these parameters except for TNF-*α* and overall data showed higher activity than Naringenin, commonly used as positive control. In general, the accumulation of triglycerides in the liver is due to an imbalance between the availability of hepatic triglycerides for export and the exporting capacity of the liver via VLDLs [34]. Furthermore, in the liver cells an increase in glucose exerts, both directly and indirectly, a series of effects which result in the orientation of its metabolism towards glycogen synthesis, glycolysis, and formation of fatty acids [6]. On the one hand a first direct effect of glucose is to stimulate its hepatic uptake which could be ameliorated with NRE activity on the lowering effect of the serum glucose level. On the other hand, an increase in triglycerides levels, particularly when accompanied by a decrease in high-density lipoprotein (HDL) levels, has been shown to be a surrogate marker of insulin resistance, a strong predisposing condition for type 2 diabetes [35].

Lipids and carbohydrates metabolism in liver is controlled by several genes. In this respect, the investigations of NRE treatment in HFD-induced obese mice on hepatic genes related to beta-oxidation, lipolysis, and lipogenesis showed a significant improvement of their expression when compared to vehicle group or Naringenin treatment. In fact, Naringenin was reported to ameliorate hepatic steatosis and attenuate dyslipidemia, without affecting caloric absorption [36] with an improvement of hepatic fatty acid oxidation through PPAR *α* coactivator 1 alpha.

Fatty acid metabolism in the liver involves three main pathways: catabolism by *β*-oxidation, synthesis, from acetyl CoA, and esterification into triglycerides. Herein in our study, NRE administration in HFD-fed mice significantly overexpressed PPAR *α* by 2 folds increase and promoted fatty acid *β*-oxidation, CPT1 gene, by 6 folds. PPAR family has been demonstrated to be highly expressed in the parenchymal cells of the liver in relation to lipid catabolism and storage. In fact, PPAR *α* is homogenous group of genes that participate in lipid catabolism such as fatty acid uptake through membrane, fatty acid binding in cells, fatty acid oxidation, and lipoprotein assembly and transport. PPAR *γ* 1 is known to influence the storage of fatty acids in the adipose tissue [37], but its mRNA expression is detected at lower level in liver. This in turn could be one of other factors (period of high-fat diet feeding…) affecting its expression in liver of mice fed with HFD compared to those fed with ND (Figure 4(a)). PPAR *α* activation is known to mediate the expression of genes promoting fatty acid *β*-oxidation mainly CPT1 gene. CPT1 is the encoding gene of carnitine palmitoyltransferase system which is a critical and essential step in the *β*-oxidation of long chain fatty acids. Such cascade of molecular events will lead finally to lowering the circulating fatty acids and triglycerides-rich lipoproteins [38]. Furthermore, NRE administration in HFD-fed mice significantly increased ACC1 gene expression encoding for the lipogenic enzyme Acetyl-CoA Carboxylase and slightly decreased FAS gene expression encoding for the fatty acid synthase at 50 mg/kg dose. Such effect demonstrates that NRE administration did not negatively affect the fatty acid metabolism since there was an enhancement of CPT1 expression in the liver of NRE-treated mice. ACC has critical roles in fatty acid metabolism and represents an attractive target for therapeutic uses in the control of obesity [39].

Regarding the gene expression of LPL that encodes the enzyme responsible for the hydrolysis of triglycerides in lipoproteins and its effect on the plasma cholesterol level, results showed that the oral administration of NRE in mice fed with high-fat diet had a highly significant overexpression of LPL more than 30-fold. In this regard, it has been demonstrated that LPL overexpression prevents the development of diet-induced hypertriglyceridemia and hypercholesterolemia and decreases VLDL and LDL fractions levels [40]. On the other hand, previous study revealed that plasma cholesterol levels were decreased in LPL transgenic mice after cholesterol loading. These findings suggest that LPL plays an important role in determining cholesterol levels. Furthermore, it has been also highlighted that free fatty acids uptake into adipocytes is also facilitated by the extracellular expression and activity of lipoprotein lipase [41]. LPL activity changes dramatically in various tissues in response to energy requirements and its systemic overexpression results in increases in whole body insulin sensitivity.

Our study demonstrated that NRE treatment in HFD fed mice significantly ameliorated the hepatic gene profile expression involved in energy homeostasis (glucose and lipid metabolism). In this respect several herbal preparations cited in the literature showed similar effects. Flavonoids like isorhamnetin [42], Naringenin [43], and quercetin [44] individually or mixed showed significant reduction in obesity and type 2 diabetes incidence.

Taken together, our results demonstrated that NRE treatment at *in vitro* and *in vivo *levels exerts antiobesity action through lowering glucose and triglycerides and the enhancement of the lipid metabolism in liver due to the increasing of serum HDL-cholesterol and the decreasing of LDL-cholesterol modulating the gene expression related to lipid metabolism. This effect may be due to the improvement of the antioxidant status within hepatic cells by the strong antioxidant activities of many phenolic components present in NRE especially flavonoids such as isorhamnetin aglycones and glycosides. Thus, the identification of possible active compounds and standardization of NRE may provide an opportunity to develop a novel class of antiobesity supplement or functional food. Further investigations will be needed in order to evaluate NRE antiobesity bioactive molecules efficacy and their bioavailability.

## Figures and Tables

**Table 1:** Composition of experimental diets.

Ingredient (%)	Normal diet (ND)	High fat diet (HFD)
Casein	18.5	25.6
L-Cystine	0.28	0.36
Maltodextrin	29.01	6.01
*α*-Corn starch	9.67	16.00
Sucrose	2.00	5.50
Soybean oil	25.00	2.00
Lard	2.00	33.00
Cellulose	6.61	6.61
Mineral mix AIN 93G	3.50	3.50
Calcium carbonate	0.18	0.18
Vitamin mix AIN93	1.00	1.00
Choline bitartrate	0.25	0.25

Total	100	100

**Table 2:** Body weight gain in ND, HFD, HFD + PC, HFD + NR50, and HFD + NR100 groups for 4 weeks.

	CND	CHFD	HFD + PC	HFD + NR50	HFD + NR100
Body weight (g)					
Initial	19.12 ± 0.40	19.18 ± 0.30	18.93 ± 0.60	19.40 ± 0.40	19.60 ± 0.40
Final	23.25 ± 0.40	27.62 ± 0.50	26.64 ± 0.20	24.60 ± 0.50*	24.40 ± 0.70*

Data represent the mean ± SEM, *P < 0.05, when compared to the CHFD group (*n* = 8 per group).

CND: control normal diet fed group.

CHFD: control high-fat diet fed group.

HFD + PC: high-fat diet + positive control (Naringenin 10 mg/kg body weight).

HFD + NR50: high-fat diet + *Nitraria retusa *50 mg/kg body weight.

HFD + NR100: high-fat diet + *Nitraria retusa *100 mg/kg body weight.

**Table 3:** Food intake (g/day) and food efficiency ratio (FER) in CND, CHFD, HFD + PC, HFD + NR50, and HFD + NR100 groups for 4 weeks.

	CND	CHFD	HFD + PC	HFD + NR50	HFD + NR100
Food intake (g)	3.25 ± 0.10	3.12 ± 0.10	3.00 ± 0.10	3.25 ± 0.20	3.06 ± 0.10
Food efficiency ratio (FER)	1.26 ± 0.07	2.70 ± 0.10	2.42 ± 0.20	1.60 ± 0.07***	1.56 ± 0.10***

Data represent the mean ± SEM, ***P < 0.001, when compared to the CHFD group (*n* = 8 per group).

CND: control normal diet fed group.

CHFD: control high-fat diet fed group.

HFD + PC: high-fat diet + positive control (Naringenin 10 mg/kg body weight).

HFD + NR50: high-fat diet + *Nitraria retusa *50 mg/kg body weight.

HFD + NR100: high-fat diet + *Nitraria retusa *100 mg/kg body weight.

**Table 4:** Blood constituents in CND, CHFD, HFD + PC, HFD + NR50, and HFD + NR100 groups after 4 weeks.

	CND	CHFD	HFD + PC	HFD + NR50	HFD + NR100
Serum total cholesterol (mg/dL)	134.00 ± 4.65	174.00 ± 8.64	154.00 ± 4.10	182.00 ± 1.55	170.00 ± 5.59
Serum HDL-cholesterol (mg/dL)	66.00 ± 4.65	80.00 ± 1.55	64.00 ± 1.55**	98.00 ± 4.10*	92.00 ± 4.10
Serum LDL-cholesterol (mg/dL)	9.00 ± 1.22	16.50 ± 2.03	6.00 ± 0.00**	18.00 ± 0.00	12.00 ± 0.00*
Serum triglycerides (mg/dL)	16.00 ± 3.11	22.00 ± 1.63	22.00 ± 1.63	10.00 ± 1.63*	14.00 ± 1.63*
Serum glucose (mg/dL)	102.00 ± 14.97	200.00 ± 4.10	146.00 ± 1.63**	168.00 ± 2.69**	153.00 ± 9.41*
Serum TNF-*α* (pg/mL)	63.20 ± 1.40	61.08 ± 0.58	61.31 ± 0.58	64.43 ± 0.56	65.72 ± 1.22

Data represent the mean ± SEM, *P < 0.05, **P < 0.005, when compared to the CHFD group (*n* = 5 per group).

CND: control normal diet fed group.

CHFD: control high-fat diet fed group.

HFD + PC: high-fat diet + positive control (Naringenin 10 mg/kg body weight).

HFD + NR50: high-fat diet + *Nitraria retusa *50 mg/kg body weight.

HFD + NR100: high-fat diet + *Nitraria retusa *100 mg/kg body weight.

## References

[1] WHO (2002). *Traditional Medicune Strategy*.

[2] Patricia M, Donohoue A (2008). *Energy Metabolism and Obesity: Research and Clinical Applications*.

[3] Dave S, Kaur NJ, Nanduri R, Dkhar HK, Kumar A, Gupta P (2012). Inhibition of adipogenesis and induction of apoptosis and lipolysis by stem bromelain in 3T3-L1 adipocytes. *PLoS ONE*.

[4] Dridi S, Taouis M (2009). Adiponectin and energy homeostasis: consensus and controversy. *Journal of Nutritional Biochemistry*.

[5] Inadera H (2008). The usefulness of circulating adipokine levels for the assessment of obesity-related health problems. *International Journal of Medical Sciences*.

[6] van den Berghe G (1991). The role of the liver in metabolic homeostasis: implications for inborn errors of metabolism. *Journal of Inherited Metabolic Disease*.

[7] Radonjic M, de Haan JR, van Erk MJ (2009). Genome-wide mRNA expression analysis of hepatic adaptation to high-fat diets reveals switch from an inflammatory to steatotic transcriptional program. *PLoS ONE*.

[8] Sahib NAG, Saari N, Ismail A, Khatib A, Mahomoodally F, Abdul Hamid A (2012). Plants’ metabolites as potential antiobesity agents. *The Scientific World Journal*.

[9] Tomoyasu Kamiya (2012). The crude extract from puerariae flower exerts anti-obesity and anti-fatty liver effects in high-fat diet-induced obese mice. *Evidence-Based Complementary Alternative Medicine*.

[10] Lee J, Jung E, Lee J (2009). Isorhamnetin represses adipogenesis in 3T3-L1 cells. *Obesity*.

[11] Hsu C-L, Yen G-C (2008). Phenolic compounds: evidence for inhibitory effects against obesity and their underlying molecular signaling mechanisms. *Molecular Nutrition and Food Research*.

[12] Ahn J, Lee H, Kim S, Park J, Ha T (2008). The anti-obesity effect of quercetin is mediated by the AMPK and MAPK signaling pathways. *Biochemical and Biophysical Research Communications*.

[13] Harmon AW, Harp JB (2001). Differential effects of flavonoids on 3T3-L1 adipogenesis and lipolysis. *American Journal of Physiology—Cell Physiology*.

[14] Ksouri R, Megdiche Ksouri W, Jallali I (2012). Medicinal halophytes: potent source of health promoting biomolecules with medical, nutraceutical and food applications. *Critical Reviews in Biotechnology*.

[15] Ksouri R, Falleh H, Megdiche W (2009). Antioxidant and antimicrobial activities of the edible medicinal halophyte Tamarix gallica L. and related polyphenolic constituents. *Food and Chemical Toxicology*.

[16] Ivanova D, Gerova D, Chervenkov T, Yankova T (2005). Polyphenols and antioxidant capacity of Bulgarian medicinal plants. *Journal of Ethnopharmacology*.

[17] le Floc'h E (1952). *Contribution a une etude Ethnobotanique de la Flore tunisienne*.

[18] El-Alali A, AlZoubi A, Gharaibeh M, Tawaha K, Alali FQ (2012). Phytochemical and biological investigation of Nitraria retusa asch. *Jordan Journal of Pharmaceutical Sciences*.

[19] Boubaker J, Ben Sghaier M, Ines S, Ghedira K, Chekir-Ghedira L (2012). Isorhamnetin 3-O-robinobioside from Nitraria retusa leaves enhance antioxidant and antigenotoxic activity in human chronic myelogenous leukemia cell line K562. *BMC Complementary and Alternative Medicine*.

[20] Boubaker J, Skandrani I, Bouhlel I (2010). Mutagenic, antimutagenic and antioxidant potency of leaf extracts from Nitraria retusa. *Food and Chemical Toxicology*.

[21] Han J, Isoda H (2009). Capsaicin induced the upregulation of transcriptional and translational expression of glycolytic enzymes related to energy metabolism in human intestinal epithelial cells. *Journal of Agricultural and Food Chemistry*.

[22] Galgani J, Ravussin E (2008). Energy metabolism, fuel selection and body weight regulation. *International Journal of Obesity*.

[23] Hadj Salem J, Chevalot I, Harscoat-Schiavo C, Paris C, Fick M, Humeau C (2011). Biological activities of flavonoids from Nitraria retusa (Forssk.) Asch. and their acylated derivatives. *Food Chemistry*.

[24] Hussein SR, Kawashty SA, Tantawy ME, Saleh NAM (2009). Chemosystematic studies of Nitraria retusa and selected taxa of Zygophyllaceae in Egypt. *Plant Systematics and Evolution*.

[25] Pandey KB, Rizvi SI (2009). Plant polyphenols as dietary antioxidants in human health and disease. *Oxidative Medicine and Cellular Longevity*.

[26] Hsu C-L, Yen G-C (2006). Inhibitory effect of phenolic acids on the proliferation of 3T3-L1 preadipocytes in relation to their antioxidant activity. *Journal of Agricultural and Food Chemistry*.

[27] Yang J-Y, Della-Fera MA, Rayalam S (2008). Enhanced inhibition of adipogenesis and induction of apoptosis in 3T3-L1 adipocytes with combinations of resveratrol and quercetin. *Life Sciences*.

[28] Aguirre L, Arias N, Macarulla MT, Gracia A, Portillo MP (2011). Beneficial effects of quercetin on obesity and diabetes. *The Open Nutraceuticals Journal*.

[29] de Santi C, Pietrabissa A, Mosca F, Spisni R, Pacifici GM (2000). Sulphation of resveratrol, a natural compound present in wine, and its inhibition by natural flavonoids. *Xenobiotica*.

[30] Liang Y-C, Tsai S-H, Chen L, Lin-Shiau S-Y, Lin J-K (2003). Resveratrol-induced G2 arrest through the inhibition of CDK7 and p34CDC2 kinases in colon carcinoma HT29 cells. *Biochemical Pharmacology*.

[31] Haider UGB, Sorescu D, Griendling KK, Vollmar AM, Dirsch VM (2003). Resveratrol increases serine15-phosphorylated but transcriptionally impaired p53 and induces a reversible DNA replication block in serum-activated vascular smooth muscle cells. *Molecular Pharmacology*.

[32] Park HJ, Yang J-Y, Ambati S (2008). Combined effects of genistein, quercetin, and resveratrol in human and 3T3-L1 adipocytes. *Journal of Medicinal Food*.

[33] Matsuda H, Kogami Y, Nakamura S, Sugiyama T, Ueno T, Yoshikawa M (2011). Structural requirements of flavonoids for the adipogenesis of 3T3-L1 cells. *Bioorganic and Medicinal Chemistry*.

[34] Lin X, Schonfeld G, Yue P, Chen Z (2002). Hepatic fatty acid synthesis is suppressed in mice with fatty livers due to targeted apolipoprotein B38.9 Mutation. *Arteriosclerosis, Thrombosis, and Vascular Biology*.

[35] Tirosh A, Shai I, Bitzur R (2008). Changes in triglyceride levels over time and risk of type 2 diabetes in young men. *Diabetes Care Journal*.

[36] Mulvihill EE, Allister EM, Sutherland BG (2009). Naringenin prevents dyslipidemia, apolipoprotein B overproduction, and hyperinsulinemia in LDL receptor-null mice with diet-induced insulin resistance. *Diabetes Journal*.

[37] Hajer GR, van Haeften TW, Visseren FLJ (2008). Adipose tissue dysfunction in obesity, diabetes, and vascular diseases. *European Heart Journal*.

[38] Maglich JM, Lobe DC, Moore JT (2009). The nuclear receptor CAR (NR1I3) regulates serum triglyceride levels under conditions of metabolic stress. *Journal of Lipid Research*.

[39] Wakil SJ, Abu-Elheiga LA (2009). Fatty acid metabolism: target for metabolic syndrome. *Journal of Lipid Research*.

[40] Yamamoto K, Shimano H, Shimada M (1995). Overexpression of apolipoprotein E prevents development of diabetic hyperlipidemia in transgenic mice. *Diabetes*.

[41] Wang H, Eckel RH (2009). Lipoprotein lipase: from gene to obesity. *American Journal of Physiology—Endocrinology and Metabolism*.

[42] Lee J, Lee J, Jung E, Hwang W, Kim Y-S, Park D (2010). Isorhamnetin-induced anti-adipogenesis is mediated by stabilization of *β*-catenin protein. *Life Sciences*.

[43] Yoshida H, Takamura N, Shuto T (2010). The citrus flavonoids hesperetin and naringenin block the lipolytic actions of TNF-*α* in mouse adipocytes. *Biochemical and Biophysical Research Communications*.

[44] Ahn J, Lee H, Kim S, Park J, Ha T (2008). The anti-obesity effect of quercetin is mediated by the AMPK and MAPK signaling pathways. *Biochemical and Biophysical Research Communications*.

